# Neurodegeneration, Neuroprotection and Regeneration in the Zebrafish Retina

**DOI:** 10.3390/cells10030633

**Published:** 2021-03-12

**Authors:** Salvatore L. Stella, Jasmine S. Geathers, Sarah R. Weber, Michael A. Grillo, Alistair J. Barber, Jeffrey M. Sundstrom, Stephanie L. Grillo

**Affiliations:** 1 Department of Neural and Behavioral Sciences, Penn State College of Medicine, 500 University Drive, Hershey, PA 17033, USA; sstella@pennstatehealth.psu.edu (S.L.S.J.); abarber@pennstatehealth.psu.edu (A.J.B.); jsundstrom@pennstatehealth.psu.edu (J.M.S.); 2 Department of Ophthalmology, Penn State College of Medicine, 500 University Drive, Hershey, PA 17033, USA; jgeathers@pennstatehealth.psu.edu (J.S.G.); sweber2@pennstatehealth.psu.edu (S.R.W.); 3 Department of Biochemistry and Molecular Biology, Penn State College of Medicine, 500 University Drive, Hershey, PA 17033, USA; mgrillo@pennstatehealth.psu.edu; 4 Department of Cellular and Molecular Physiology, Penn State College of Medicine, 500 University Drive, Hershey, PA 17033, USA

**Keywords:** light-induced retinal degeneration, chemical toxicity, mechanical damage, oxidative stress, diet, retinal stab, retinal damage, optic nerve, photoreceptors, retinal ganglion cells

## Abstract

Neurodegenerative retinal diseases, such as glaucoma and diabetic retinopathy, involve a gradual loss of neurons in the retina as the disease progresses. Central nervous system neurons are not able to regenerate in mammals, therefore, an often sought after course of treatment for neuronal loss follows a neuroprotective or regenerative strategy. Neuroprotection is the process of preserving the structure and function of the neurons that have survived a harmful insult; while regenerative approaches aim to replace or rewire the neurons and synaptic connections that were lost, or induce regrowth of damaged axons or dendrites. In order to test the neuroprotective effectiveness or the regenerative capacity of a particular agent, a robust experimental model of retinal neuronal damage is essential. Zebrafish are being used more often in this type of study because their eye structure and development is well-conserved between zebrafish and mammals. Zebrafish are robust genetic tools and are relatively inexpensive to maintain. The large array of functional and behavioral tests available in zebrafish makes them an attractive model for neuroprotection studies. Some common insults used to model retinal disease and study neuroprotection in zebrafish include intense light, chemical toxicity and mechanical damage. This review covers the existing retinal neuroprotection and regeneration literature in the zebrafish and highlights their potential for future studies.

## 1. Introduction

Neuroprotection is the process of preserving the structure and function of neurons from a harmful insult, such as a toxic compound or a neurodegenerative disease. This is often achieved by applying a pharmacological or non-pharmacological (natural or genetic) agent to a neuronal population, such as the retina, preceding or during a neurological insult. The same retinal injury paradigms used for neuroprotection are also used to determine mechanisms of neurodegeneration and regeneration. Retinal neurodegenerative diseases, such as glaucoma and diabetic retinopathy, result in a loss of neurons in the retina as the disease progresses, which often leads to vision loss. Central nervous system (CNS) neurons are not able to regenerate in mammals, therefore, an effective neuroprotective strategy to protect or regenerate retinal neurons during disease progression is urgently needed. Low-cost and high-throughput screening techniques to identify drug or genetic targets for retinal protection or regeneration are essential. Hence, the small size, low cost, and robust genetic tools of zebrafish make them ideal for this type of screening analysis. More importantly, their vision is cone-dominated, like humans, and their eye structure and development is well-conserved across vertebrate species. Consequently, more researchers have turned to zebrafish to study retinal neurodegenerative diseases. Additionally, zebrafish retinas have the ability to regenerate after some injuries, and offer a unique opportunity to examine regenerative mechanisms that could ultimately contribute to the development of neuroprotective and regenerative therapies in humans.

There are several detailed reviews that have been published within the last 10 years describing the extensive toolkit (transgenic models, antibodies, ERGs and visually guided behaviors) available for conducting vision research in zebrafish [1,2,3,4,5]. Here, we highlight the innovative studies that have employed the use of zebrafish to study and develop neuroprotective and regenerative strategies and targets for retinal diseases. We have attempted to summarize current literature regarding retinal neuroprotection and regeneration in zebrafish retinal injury paradigms.

## 2. Clinical Significance of Neurodegeneration in the Retina

Vision loss in retinal diseases occur by a diverse array of mechanisms which often culminate in cell death of retinal neurons. Neurodegeneration is thus a common feature underlying irreversible vision loss in retinal disease, and substantial evidence points to its critical role in inherited retinal dystrophies and optic neuropathies. Neurodegeneration manifests clinically as morphological and functional changes including thinning of the outer nuclear layer and degeneration of the ellipsoid zone detected by optical coherence tomography (OCT), correlating to visual deficits apparent on visual field or electroretinogram (ERG) testing [6,7,8]. The clinical significance of retinal neurodegeneration, and lack of therapeutic options, emphasizes the importance of animal models such as zebrafish, which allow us to elucidate disease mechanisms and uncover potential targets for therapeutic intervention.

### 2.1. Inherited Retinal Dystrophies

Inherited retinal dystrophies (IRDs) refer to a heterogeneous group of hereditary diseases characterized by progressive dysfunction and degeneration of retinal cells, initially involving photoreceptors, with subsequent loss of inner retinal cells. Surprisingly, the inner retina stays intact for a remarkably long period of time following outer retinal degeneration or injury, which inspired many restorative approaches of outer retina cells [9,10,11]. IRDs encompass retinitis pigmentosa (RP), a subgroup of these disorders that affects 2 million people worldwide [12] with a prevalence of 1 per 750 to 9000 individuals, depending on the region in which data were gathered [13,14]. Over 3000 different mutations in about 70 genes have been identified as causes of RP [14]. Much of the evidence of neurodegeneration in IRDs derives from animal models, including mice and zebrafish, with mutations similar to those that occur in human forms of the disease. From the patient perspective, these changes typically manifest as nyctalopia and progressive constriction of peripheral vision. IRDs as a group were previously untreatable. Recently, however, the Food and Drug Administration approved gene therapy for the treatment of RPE65-related Leber congenital amaurosis in patients with *RPE65* mutations after gene replacement demonstrated improvement of functional vision [15]. Clinical trials investigating similar treatments for IRDs caused by other genetic mutations are underway, but it should be emphasized that treatment of IRDs remains severely limited.

### 2.2. Optic Neuropathies

Glaucoma is a category of eye diseases classically marked by elevated intraocular pressure in which retinal ganglion cell (RGC) death leads to irreversible blindness. Globally, glaucoma affects nearly 80 million people [16,17,18] and is the second leading cause of blindness, after cataracts [19]. Neurodegeneration in the form of RGC death is well documented in glaucoma [20,21], and recent evidence demonstrates that RGC axon degeneration extends into the brain [22]. Treatment of the most common forms of glaucoma is largely aimed at keeping intraocular pressure within an acceptable range. This can be accomplished by administering topical agents that decrease aqueous humor production or increase its outflow, such as prostaglandin analogs, beta-adrenergic antagonists, alpha-adrenergic agonists, parasympathomimetic agents, and carbonic anhydrase inhibitors. Interestingly, some of these medications appear to be neuroprotective (e.g., prevent RGC death) independently of their effects on intraocular pressure [23]. If medical therapy fails to sufficiently manage intraocular pressure, laser therapy or surgical interventions can be considered. Treatment is mainly aimed at stabilizing vision, because once vision loss occurs it cannot be recovered [20].

Like glaucoma, optic neuritis also involves RGC damage, but results from an inflammatory, demyelinating process, often in association with multiple sclerosis [24,25], though the disease may also result from other inflammatory conditions, infections, toxicities, or is idiopathic in nature [26]. The annual incidence of optic neuritis in the US is estimated at 6.4 per 100,000 [25,27], with many of these patients being left with visual deficits after recovery [28]. In the Optic Neuritis Treatment Trial, about 40% of patients experienced permanent vision loss [29]. Thinning of the nerve fiber layer, which is presumed to represent the loss of RGC axons, has been shown to correlate with visual deficits following optic neuritis episodes [30,31,32], suggesting that vision loss in optic neuritis results from a neurodegenerative process. Optic neuritis presents clinically as a monocular, painful vision loss, which typically improves over a period of weeks to months. Administration of corticosteroids accelerates recovery of vision but does not appear to benefit long-term visual acuity or visual field [29].

Diabetic retinopathy is a complication of diabetes characterized by progressive microvascular lesions in the retina. Though typically asymptomatic in its early stages, diabetic retinopathy confers a significant risk of vision loss in later stages. The prevalence of diabetic retinopathy increases as the duration of diabetes increases [33], and its prevalence among diabetic adults over 40 years of age is estimated at 28.5% in the US and 34.6% globally [34,35]. Although diabetic retinopathy has traditionally been viewed as a primarily microvascular disease, it is now recognized that neurodegeneration precedes these changes and plays a critical role in diabetic retinopathy pathogenesis [36,37,38]. Neuronal apoptosis, reactive gliosis, and changes in the expression of glial fibrillary acidic protein (GFAP) are all features of neurodegeneration that have been observed in animal models of diabetic retinopathy [39,40,41]. In line with these findings, thinning of the nerve fiber and ganglion cell layers have been observed in patients with pre-clinical diabetic retinopathy, and this phenomenon was similarly observed in animal models of both type 1 and type 2 diabetes [42]. Additionally, a recent proteomic study of human retinas from patients with pre-clinical diabetic retinopathy identified several pathways involved in neuroprotection and neurodegeneration [43], further substantiating neurodegeneration as an early component of the disease. Clinically, acute vision loss in diabetic retinopathy often arises secondary to microvascular complications, but permanent vision loss following treatment and resolution of these complications suggest that neurodegeneration may contribute to the loss of function. Management of diabetic retinopathy in the early stages of disease focuses on controlling other aspects of diabetes, such as blood pressure and levels of lipids and glucose. In later stages of disease, intravitreal anti-vascular endothelial growth factor injections may be indicated to mediate neovascular aspects of the disease. There are currently no treatments available that directly target the neurodegenerative changes of diabetic retinopathy. Diabetes can be modelled in zebrafish, at least under acute circumstances, and there is opportunity to use this model to specifically explore the effects of hyperglycemia on cone-mediated vision.

Optic nerve injury, also referred to as traumatic optic neuropathy can arise from direct or indirect mechanisms, usually via orbital laceration or optic nerve contusion, respectively [44]. In a study conducted at a large Canadian trauma center, optic nerve injury accounted for 0.4% of all trauma patients [45]. In mammalian studies using optic nerve transection, RGCs survive for several days following injury then collectively undergo apoptosis [46,47,48]. The RGC population is reduced to only ~10% of their original population after two weeks post-transection [46,49,50,51]. However, it is important to note that the rate of RGC degeneration after a traumatic optic neuropathy is dependent on the distance of the injury site from the eye or cell soma [52]. Thus, neurodegeneration appears to be a pronounced sequela of both direct and indirect optic nerve injury. There are no strong recommendations for treatment intervention in optic neuropathy, as treatments such as corticosteroid administration do not improve visual acuity outcomes relative to observation without pharmaceutical treatment [53].

Currently, there are no treatment options available for restoring vision loss in retinal neurodegenerative diseases, highlighting the need for more extensive research using animal models, including zebrafish.

## 3. The Use of Zebrafish for Retinal Neuroprotection and Regeneration Studies

### 3.1. Advantages of the Zebrafish Animal Model

Zebrafish (*Danio rerio*) serve as an excellent model system to study the vertebrate visual system, including circuitry, behavior, and disease. Zebrafish have a diurnal circadian rhythm, are cone-dominated, have good color vision, and relatively high visual acuity. In contrast, mice and rats, which are the predominant animal models used in vision research, have a nocturnal circadian rhythm, rod-dominated retinas, poor or absent color vision, and relatively low photopic visual acuity (especial in the albino strains). Zebrafish display robust visual behaviors including optokinetic reflexes, optomotor guided visual responses, visual-motor startle responses, phototactic or light-driven visual behaviors, and a visually guided escape response. This makes testing for changes in vision and visual behavior in zebrafish relatively easy to learn and perform, and highly accessible for either the animal or the tester. Another remarkable similarity between zebrafish and mammals is the common signaling pathways underlying CNS regeneration [54,55,56,57]. Considering the structural and functional similarities shared with the human retina, zebrafish may be a more appropriate model for studying human ocular diseases, particularly those that affect cone photoreceptors such as age-related macular degeneration and diabetic retinopathy.

### 3.2. The Zebrafish Visual System

Zebrafish are a well-established vertebrate animal model that is widely used for the study of visual behavior, circuitry, and disease. The zebrafish retina is comparable to most classes of vertebrates in terms of development, structure, and function. While mice and rats have been widely used to study vision and visual behavior, zebrafish have a slight advantage, whereby their retinas are similar to human retinas in many ways and are in some respects a better model for studying vision.

**Figure 1:**
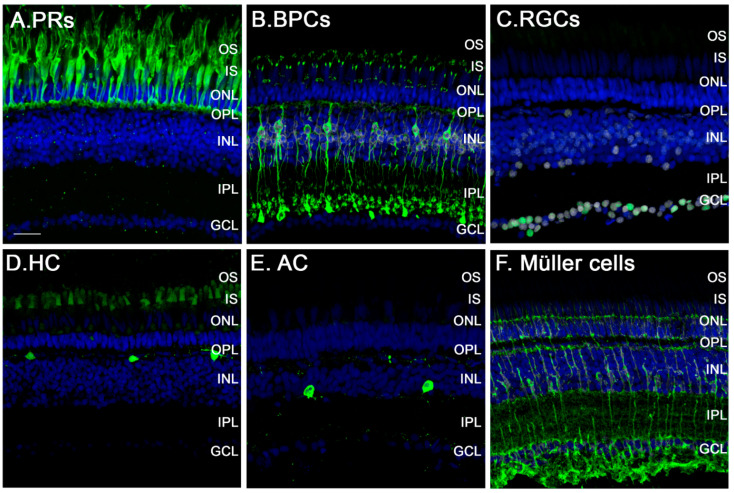
Neurons and glia in the zebrafish retina. Confocal image showing examples of neurons and glia (green) in the zebrafish retina: (**A**) anti-Zpr1 labels the double-cone (DC) photoreceptors; (**B**) anti-PKCα labels the large Mb-1 bipolar cells and all ON-bipolar cells; (**C**) anti-islet1/2 labels the RGCs, (although a few other cell types are labelled non-specifically in the INL); (**D**) transgenic Lhx1A zebrafish have GFP-labelled horizontal cells in the OPL; (**E**) anti-tyrosine hydroxylase labels the amacrine cells in the INL; (**F**) anti-glutamine synthetase to label the Müller glia. Nuclei are also labeled (blue). Other glial cells that are present in the zebrafish retina include microglia and astrocytes (not labeled). Abbreviations: PRs, photoreceptors; BPCs, Bipolar cells; RGCs, retinal ganglion cells; HC, Horizontal cells; AC, Amacrine cells; OS, outer segments; IS, inner segments; ONL, outer nuclear layer; OPL, outer plexiform layer; INL, inner nuclear layer; IPL, inner plexiform layer; GCL, ganglion cell layer. Scale bar = 20 µm.

The human retina has a specialized cone-dense area responsible for central high-resolution color vision known as the macula. Although, the macula is absent from both the rodent and zebrafish retina, unlike mice and rats, zebrafish are diurnal, meaning that they are active during the day and sleep at night. Consequently, the retinas of zebrafish are cone-dominated (Figure 1A) [58] and they utilize their color vision [3,58]. During daytime conditions, zebrafish rely heavily on color vision to locate food, avoid predators, and move around throughout their underwater environment. Therefore, while zebrafish do not have a macula, they do have a cone-dominated retina which is akin to the human macula resulting in good color vision and a cone density similar to humans [58]. In contrast, mice and rats are nocturnal, meaning that they are most active during the night. Rodent retinas are rod-dominated with rods making up about 97% of the photoreceptors and cones making up the remaining 3%, resulting in relatively poor color vision [59]. In order to navigate their dark environment mice rely more on olfactory, tactile, and auditory cues rather than vision [60,61,62].

**Figure 2:**
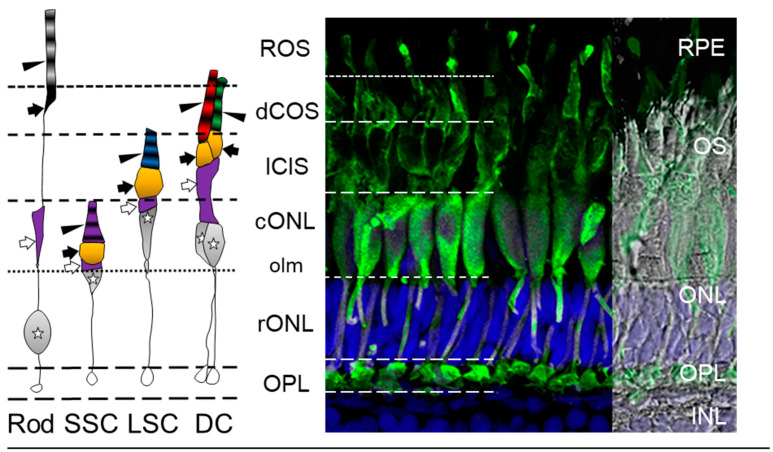
Types of photoreceptors in the zebrafish retina. Illustration showing the different types of photoreceptors in the zebrafish outer retina, along with the location of their outer segment (black arrowhead), ellipsoid (black arrow), myoid (white arrow), nuclei (star), and terminals in the OPL. Confocal image shows anti-Zpr1 (green) labelling of DC photoreceptors and nuclei (blue) in the zebrafish retina. Abbreviations: SSC, short single cones (SSC); LSC, long single cones; DC, double cones; ROS, rod outer segments; dCOS, double cone outer segments; LCIS, long cone inner segment sublayer; cONL, cone outer nuclear layer; olm, outer limiting membrane; rONL rod outer nuclear layer; OPL, outer plexiform layer, RPE, retinal pigment epithelium; OS, outer segment; ONL, outer nuclear layer; INL, inner nuclear layer. Figure adapted from [64].

Similar to the human, zebrafish have a retina that is composed of three nuclear layers (ONL, INL, GCL) separated by synapse-rich plexiform layers (OPL, IPL). The zebrafish retina contains the same broad classes of retinal neurons (photoreceptors (Figure 1A), bipolar cells (Figure 1B), RGCs (Figure 1C), horizontal cells (Figure 1D), and amacrine cells (Figure 1E)) as well as glial elements (Müller cells (Figure 1F) and astrocytes, microglia) [3,63]. The ONL of the zebrafish contains a single type of rod and four spectral and morphological types of cones making them tetrachromatic (Figure 2). The rod cell bodies (rod ONL [rONL]) are located superficially to the cone nuclei (cone ONL [cONL]); and in the light-adapted retina the rod inner and outer segments (rOS) project beyond the cones to interdigitate with the microvilli of the retinal pigment epithelium (RPE) [63].

In a cross section of the zebrafish retina, the cone photoreceptors are tiered within the ONL and are organized morphologically into: short single cones (SSC, ultraviolet-sensitive cones); long single cones (LSC, blue-sensitive cones) above the short single cones; and, lastly, the double cones (DC), consisting of a principal red-sensitive (L) cone and accessory green-sensitive (M) cone [3,65]. Zebrafish possess genes for a single blue opsin (SWS2), a single ultraviolet (UV) opsin (SWS1), two forms of red opsins (LWS-1 and LWS-2), and four forms of green opsins (RH2-1, RH2-2, RH2-3, RH2-4) [66]. In addition, cones can be classified based upon their peak spectral sensitivities of their expressed opsin proteins, present in each type of cone: UV-cones λ_max_ = 355 nm (SWS1); blue-cones λ_max_ = 416 nm (SWS2); red-cones λ_max_ = 558 nm (LWS-1) and 548 nm (LWS-2); and green-cones λ_max_ = 467 nm (RH2-1), 476 nm (RH2-2), 488 nm (RH2-3), and 505 nm (RH2-4) [66]. In the adult zebrafish retina, cones appear in a mosaic pattern where columns of alternating short single cones and long single cones alternate with columns of red-green double cones [67]. Humans, in contrast, have trichromatic vision and lack UV-cones, while mice are dichromatic and have two types of cones: co-expressing cones, single cones that express both S and M- cone pigments, and a small population of true S-cones which allows color vision [59,68,69,70,71]. However, recently, these true S-cones have been described as highly concentrated in the ventral retina [72] and provide the ability to distinguish color [73]. That specific distribution is reminiscent of the UV-cone distribution in the larval zebrafish retina, but with different functionality [72,74]. In mice, co-expressing cones show a gradient of mostly M-opsin (green) expression in the dorsal retina to mostly S-opsin (blue/UV) expression in the ventral retina [70]. Thus, the small percentage (~3%) of mouse cone photoreceptors co-expressing M- and S-cone opsins in a unique dorsal-ventral gradient likely confers very poor visual acuity in the visible light spectrum, and non-existent color vision, whereas the zebrafish color vision is more comparable to that of humans.

### 3.3. Zebrafish Visual Acuity

In addition to good color vision, zebrafish also have relatively high visual acuity. In Caves et al. (2018), the authors define visual acuity as the ability of an animal to perceive static spatial detail. The authors explain that visual acuity tends to be higher in diurnal animals than in nocturnal animals like mice and rats. This is because the retina of nocturnal animals frequently exhibits spatial summation, a process where the photoreceptors pool together to collect light over a larger area making them functionally a single sampling unit, which is more often tied to rods and rod dominated species. While this increases the sensitivity and is presumably more adaptive for low-light situations, it comes at the cost of visual acuity. Furthermore, visual acuity tends to be higher in species that live in spatially complex habitats such as an underwater environment. Overall, humans have exceptionally high visual acuity that is only surpassed by a few predatory bird species [75].

Zebrafish visual acuity is typically measured using behavioral tests, (e.g., the optokinetic response and optomotor response), however another excellent measure of visual acuity can be tied to the density of RGCs in the retina [76,77]. Areas of the retina with a greater density of RGCs have been shown to have a higher visual resolution and animals with greater overall mean RGC density tend to have higher visual acuities [77,78,79]. The average RGC density in the zebrafish retina ranges from 11,960 ± 4095 cells/mm^2^ to 37, 245 ± 7055 cells/mm^2^ [80].

The peak density of RGCs for zebrafish was found in the ventral-temporal retina, while the lowest density of RGCs occurred in the dorsal-nasal region of the retina resulting in ~300% increase in RGCs from the peripheral to central retina. In contrast, the average RGC density in rats ranges from 600 cells/mm^2^ to 3600 cells/mm^2^ [81]. Danias et al. 2002, in an original publication, did not describe a consistent pattern of higher RGC density in rat retina [81], however, more recent publications support the idea of a higher RGC density in the dorsal [82] or dorso-central retina above the ON head in rats [50,82,83] and similarly, but less evident, in mice [48,51,84]. These RGC density differences suggest that the zebrafish may be a superior model of inner retina structure and function compared to the rodent. The density of photoreceptors have also been correlated to visual acuity in larval retina, with areas of higher photoreceptor density having higher visual acuity as concordant with Nyquist-Shannon sampling theorem, which describes the ultimate limit of visual acuity as distance between photoreceptor [85]. The density map of the photoreceptor differ between types and match behavioral demands, such as catching prey [74].

### 3.4. Müller Glia: Source of Regenerated Neurons in the Retina

The two populations of stem cells in the zebrafish retina are (1) multipotent retinal progenitors located in the germinal zone or ciliary marginal zone (CMZ), which add cells to the peripheral retina as the fish grows, and (2) the radial glia (Müller cells), which can undergo asymmetric division to generate committed progenitors [86,87,88,89]. When the zebrafish retina is damaged, the regenerative response is initiated and the asymmetric cell division of Müller glia to produce neuronal progenitor cells [86,90,91]. These cells then proliferate and migrate to the site of injury to differentiate into the required neuronal cell type [90,91]. In 2006, Fausett et al. showed that Müller glia expressing α1T, a neuron-specific microtubule protein induced in the developing and regenerating CNS, can de-differentiate and become multipotent in the injured zebrafish retina [92]. Shortly after, Bernardos et al. (2007) found that Müller glia also express the multipotent progenitor marker Pax6 (paired box gene 6), and have a low level of proliferation in the uninjured retina. Müller glia-derived progenitors express Crx (cone rod homeobox), and are late retinal progenitors, and remain competent to regenerate missing retinal neurons [93]. The same group later showed evidence for asymmetric, self-renewal divisions of injury-induced Müller glia and that N-cadherin-mediated adhesion is required in the regeneration of neurons [89]. In 2007, Fimbel used intravitreal injections of oubain to demonstrate that inner retinal damage is sufficient to induce regeneration of the INL and ganglion cells in the zebrafish retina [88]. They also showed later that transcription factors, Pax6 and Ngn1, and transgene, olig2: EGFP can be used to identify different cell types at particular stages of retinal regeneration [87].

## 4. Robust Endpoints for Retinal Neuroprotection Studies in Zebrafish

### 4.1. Behavioral Paradigms

A number of studies have developed behavioral paradigms to effectively assess visual function in zebrafish, utilizing visual reflexes and hunting behavior, and these approaches have been reviewed previously [94,95]. The visual behaviors of both larval and adult zebrafish are easy to assay and require no training for the fish. There are a variety of robust assays that can be used to study the zebrafish visual system and test for visual deficits. The most commonly used approaches to assess visual function rely on the optomotor response (OMR), optokinetic reflex (OKR), the startle response (SR), and the phototactic behavioral response [94].

#### 4.1.1. Optokinetic/Optomotor Response (OKR/OMR)

The OMR and OKR provide evidence of a fully functioning visual system, and serve as a measure of visual acuity and contrast detection. The OKR is based on reflexive eye movements in response to a lateral moving stimulus, such as a rotating black and white grating, in order to help stabilize the image on the retina [63]. The OKR relies only on tracking eye movements of the zebrafish, so both larval and adult zebrafish are immobilized during these tests. The OKR develops between 73 and 80 h post-fertilization (hpf) and persists throughout adulthood [94]. The OMR is similar to the OKR but the stimulus drives head and body movement rather than the eyes. In zebrafish, the OMR is the tendency of a fish to swim towards a moving stimulus and involves the coordination of central circuitry along with both head and body movements [3]. The OMR in zebrafish develops later than the OKR, at 5 days post fertilization (dpf), can be reliably evoked at 7 dpf, and will persist throughout adulthood [94]. Zebrafish with visual deficits will swim in random patterns in the case of the OMR assay, or will be unable to track the moving stimulus with their eyes in the case of the OKR assay. The OKR (and to a lesser extent the OMR) was initially used as a rapid visual assay to identify zebrafish mutations with visual defects. Many mutants that were identified by this approach are still studies today.

The OKR is mediated by sub-cortical neural pathways, and does not require higher visual cortical processing, so is considered to be primarily a measure of sensory input and neuronal efficiency within the retina. Behavioral paradigms utilizing the OKR do not require learning trials, and again this is an advantage because the OKR is not influenced by neural changes at the cortical level. It has been recognized for some time that the zebrafish has a robust OKR that can be used to assess visual function [96]. The zebrafish OKR has high resolution and is primarily a cone-driven response [97]. It is also sensitive to drugs that are similarly toxic to vision in humans, suggesting that the zebrafish OKR can be used to predict oculotoxicity in humans [98]. Detecting the OKR is a convenient approach to measure visual function in awake animals and is very effective when used in combination with genetic disease models in the zebrafish. For example, the OKR was used to confirm functional deficits due to progressive selective degeneration of cone photoreceptors in the *eclipse (els)* zebrafish, which carries a mutation of the cGMP phosphodiesterase 6α′ subunit [99]. Similarly, morpholino knock-down of *unc119c*, which is involved in G-protein trafficking in photoreceptors and is associated with cone-rod dystrophy, leads to functional cone-selective degeneration confirmed by loss of the OKR, possibly by upregulation of the brain derived neurotrophic factor-TrkB signaling axis [100]. The OKR was also used to confirm functional vision loss in *rbpr2^−/−^* mutant zebrafish, which have extensive outer retina and RPE degeneration, presumably because loss of the all-trans-retinol receptor causes a failure of vitamin A uptake into the retina [101]. The OKR can also be used to determine pharmacological effects on vision, including testing potential treatments for diseases of vision. The OKR saccade frequency, for example, proved to be a sensitive method to test the effectiveness of histone deacetylase inhibitors to rescue vision in the *dye^ucd6^* zebrafish, which broadly model inherited retinal degenerations [102].

The OKR has been used as a powerful approach to link neuronal dysfunction with anatomical measures of neurodegeneration and recovery. The zebrafish retina has the ability to recover rapidly from certain neurodegenerative injuries and offers a method to understand mechanisms of synaptogenesis and neurogenesis that could be applied to future development of neuroprotective therapeutics. Visual acuity can be reduced in adult zebrafish over a three-day period by the addition of N-methyl-N-nitrosurea, which induces photoreceptor degeneration. This treatment initially causes vision loss but the behavioral response gradually recovers over a 25-day period, and there is a matching histological recovery in the retina, demonstrating that zebrafish neurogenesis results in functional recovery of vision [103]. OKR was also used to confirm loss of function after conditional ablation of blue and ultraviolet cone photoreceptors, and the rapid recovery of visual function suggests a robust potential for compensatory synaptogenesis in the zebrafish retina [104].

The OKR response is sensitive to retinal network mutations that may not affect the ERG output in a measurable way, and is likely derived from inner retina processing. Support for this comes from the *Celsr3* cadherin mutation, which causes zebrafish to have a supra-physiological ERG b-wave amplitude, possibly due to elevated expression of bipolar cell GABA receptors, but the OKR in these fish is significantly reduced, indicating loss of functional vision. This study suggests that retinal output that enables a normal OKR is dependent on appropriate inner retina signal processing determined by normal ON-pathway signaling [105]. The OKR deficits in contrast sensitivity also become significant in the *bugeye* mutant, a model of glaucoma, at least two months before the b-wave amplitude of the ERG is reduced, further indicating the value of the OKR to assess vision loss compared to the ERG [106].

Perhaps the most revealing studies on the OKR have utilized the transparent brain of larval zebrafish to image neural activity during the OKR response, thus mapping entire neural circuits related to visual function. Two-photon imaging of zebrafish larvae expressing the GCaMP5G Ca^2+^ indicator revealed that four temporal and symmetrical clusters of neurons become active during OKR behavior, suggesting that this simple reflex involves a widely distributed network of neurons in the brain [107,108]. This powerful whole-brain imaging approach could be used in combination with retinal neurodegeneration mutations to determine their effects on brain development and information processing.

#### 4.1.2. Startle Response (SR)

The SR is a defensive reflex designed to cause the zebrafish to escape danger such as sudden movement by a predator, and can be triggered by rapid changes in the environment, i.e., tactile, acoustic, or visual stimuli. The SR causes the zebrafish to avoid dark areas, moving objects, and open spaces [109]. The mechanism underlying this effect in zebrafish is dependent upon the size and speed of the stimulus, with brighter stimuli eliciting more robust responses [94,109]. Contractions of the axial musculature leading to increased swimming movement can be initiated by both acoustic and visual stimuli [110]. The SR is relayed through bilateral giant neurons called Mauthner cells (M-system), which are components of the Brainstem Escape Network that likely receive this startle-related information from the tectum, and are influenced by both auditory and visual stimuli [94,111]. Specialized RGCs innervate tectal superficial inhibitory neurons [112,113], that alter the activity of tectal neurons relaying their information, directly or indirectly, to the M-system, which triggers the response following light onset or light offset [94,109,113]. Thus, the visual startle reflex is based on a sudden exposure to light that triggers body movement through the M-system and provides a mechanism for escape behavior [3]. The response is also regulated through bilateral directionality of movement because the SR is determined by comparative luminance detection at the level of the thalamus [114]. This visual pathway is also sensitive to ultraviolet stimuli [115].

The SR reflex has been used to assess the effectiveness of the visual system, separate from using the OKR, using video tracking of locomotor activity during 30 min lights-on and lights-off periods of luminance, allowing light or dark adaptation to occur during each period. The rapid transition between different light intensities triggers a change in locomotion followed by a gradual return to baseline activity. Sudden light onset causes a reduction in locomotion while the light termination induces a rapid increase in movement [116].

It is likely that the startle response initiated by visual stimuli is more robust than the OKR reflex. The *no optokinetic response c* (*nrc*) zebrafish mutation was found to completely abolish the OKR and this fish was initially thought to be blind [117]; however, a further study showed that *nrc* fish retained a partially normal SR, and continued to respond to the light-OFF stimulus while having a significantly reduced, and delayed light-ON response [116]. It is likely that the partial behavioral deficit is due to retention a normal OFF-RGC signaling pathway, while the ON-RGC pathway is significantly compromised in the *nrc* fish [118].

The SR relay system is a useful model to study mechanisms controlling axon growth in development. A mutation in the *Robo* receptor causes axon misguidance of Mauthner cells resulting in their failure to cross the midline. The consequent loss of the unidirectional SR reveals the functionality of these cells [119]. Non-associative habituation learning paradigms have also been developed based on the modulatory response to repetitive visual stimuli similar to the SR. This behavioral paradigm of memory has been used to establish that zebrafish show robust short-term habituation as well as protein synthesis-dependent long term memory [120]. Changes in Ca^2+^ dynamics in Mauthner cells indicate that short-term habituation is regulated at the level of dendritic excitability [121]. Development of the Mauthner cell is depended on the zebrafish homologue of amyloid precursor protein, called appb. Antisense morpholino knock-down of appb abolished electroshock escape behavior, which presumably works through a similar path as the SR. It also caused developmental abnormalities in the structure of Mauthner cells [122]. These studies offer a potential approach to study some of the molecular mechanisms of Alzheimer’s disease. Mauthner cells are also incapable of regenerating, so this model can be used to study molecular mechanisms of spinal cord injury, with locomotor activity as a functional outcome measure [123].

The SR offers a simple behavioral paradigm that can be used to assess the impact of genetic or pharmacological induction of neurodegeneration of the visual system and visuospatial motor relays. The convenience of genetic manipulation and the ability to automate locomotor tracking in zebrafish suggest that this model has great potential for further development.

#### 4.1.3. Phototaxis or Phototactile Behavioral Response

Zebrafish, as described above, use sensory cues to navigate towards environments where they are more likely to avoid predators, obtain food or find mates. Similar to the OMR, efficient goal-directed locomotion requires coordination between motor action and sensory perception. In the visual system of larval and adult zebrafish this is accomplished through phototaxis or the phototactile behavioral response. This behavior drives zebrafish towards illuminated regions and is hard-wired in the zebrafish visual system at 5 dpf [124]. The phototactic behavioral response test utilizes the tendency of the zebrafish to move towards an illuminated chamber [3]. In this assay, larval or adult zebrafish are placed in a rotating white drum with a single black strip that simulates a threatening predator. Fish with visual deficits will swim in random patterns and not display the predator-avoidance response [94,109].

### 4.2. Retinal Imaging

The technology originally designed to image patient retinas in the clinical setting, for diagnostic and research purposes, is now being adapted for use in the laboratory setting. The most commonly used technology is spectral domain optical coherence tomography (SD-OCT) which can provide cross-sectional and en face images of rodent and fish eyes, usually at even higher resolution than those generated in the clinical setting.

#### Optical Coherence Tomography

Spectral domain optical coherence tomography (SD-OCT) retinal imaging has become an important diagnostic approach in the clinic. Equally, it is a valuable non-invasive imaging tool for basic research. SD-OCT devices used to image rodent retinas have been further adapted to study the zebrafish retina in high resolution. Due to the small size and optical properties of the zebrafish eye, en face images of the photoreceptor matrix can be produced at a single-cell resolution that is not possible with rodent and human eyes [125,126]. Quantification and tracking of individual cones is highly reproducible using this approach [127]. Voroni domain analysis has also been used to determine the cone-domain packing regularity and how this gradually decreases as the zebrafish aged from 3 to 12 months old [128].

Recent studies have employed SD-OCT in zebrafish models of degenerative disease. In laser-induced retinal neovascularization, which is used to model wet age-related macular degeneration, SD-OCT was used in conjunction with histological and immunohistological analysis to assess Müller cell activation [129]. Outer retina hyperreflectivity resulting from the laser induced injury could be detected by SD-OCT and was similar to that of rodent models. The in vivo imaging approach showed that the regenerative response to injury took place over a 14-day period and corresponded to Müller cell expression of GFAP, detected by immunohistochemistry, suggesting that the macroglial inflammatory response plays a role in wound recovery and regeneration in the zebrafish retina. The non-terminal nature of the in vivo imaging studies can be effectively combined with behavioral assessment of visual function. A recent study used SD-OCT to track retinal hemorrhage and repair after a needle-induce retinal lesion and also measured functional change within the same fish using a T-maze choice chamber paradigm [130]. The study showed a very close correlation between the extent of retinal hemorrhage and performance of the T-maze, suggesting that mechanical damage impacts visual function, and that both retinal structure and function are recoverable in a 14–15-day period after this type of injury.

While there are currently only a small number of investigators using SD-OCT to study the zebrafish retina, it appears that the zebrafish eye offers an opportunity for high resolution in vivo imaging that allows for detailed mapping of cone receptors as well as convenient measurement of neural injury and recovery in ways that are not available in rodent models.

### 4.3. Functional Endpoints

#### 4.3.1. Electroretinography

In addition to using behavioral assays to study the zebrafish visual system, retinal function and neurotransmission can be studied using the electroretinogram (ERG). The ERG is an electrophysiological method of field recording that can be used to measure electrical activity of cells in the retina in response to a brief flash of light [131]. When visual input is detected and processed in the retina, an electrical field potential is generated and transferred to the corneal surface, where it can be detected. Unlike the OMR, which captures visual function across the CNS as a whole, the ERG assesses neural function specifically in the retina. The ERG has been considered a test of retinal function both clinically and experimentally for many years. A great advantage of the ERG is how it translates between different species, since the classic ERG waveform is basically the same in humans, rodents and fish. While the small size of the zebrafish eye makes ERG measurements technically difficult, modern electrodes and amplifiers have made it possible to collect ERG recordings comparable with the larger species of animals [132]. In zebrafish, three major components are generally used to assay for visual deficits: (1) the a-wave, which is a measure of the photo-response or activity of photoreceptors; (2) the b-wave or ON response, which is a measure of photoreceptor to ON-bipolar cell neurotransmission; and (3) the d-wave or OFF response, which is measured at light offset with longer periods of light stimuli (≥0.5 msec) [133]. It is possible to record all three waveforms using a thin glass pipette carrying a chloride/silver wire electrode and Ringers solution, inserted near the cornea of anesthetized fish, under both photopic and scotopic conditions [134]. It is even possible to record ERG b-waves from isolated zebrafish eyes, and this technique has been used to examine the contribution of acetylcholine on the ERG waveform by bathing the entire eye in nicotine solution [135]. More recent technological innovations include using LED stimulators [136], and use of a sponge-tip electrode in place of the standard glass pipette [137].

The ERG first appears as early as 4 dpf in zebrafish, with measurements of the a-wave, b-wave, and d-wave, however only cones contribute to early larval ERG measurements with rod function appearing at later stages of development closer to adulthood [133]. The ERG has been used in zebrafish models to quantify the degree of retinal neurodegeneration in a variety of models of inherited retinal diseases. In a model of night blindness, in which there are varying degrees of rod outer segment degeneration, the ERG b-wave amplitude correlates with both the loss of visual function and histological parameters, suggesting that the ERG is a valid measure of neurodegeneration in zebrafish [138]. The myosin 7aa^−/−^ mutation in zebrafish is thought to model Usher syndrome in humans, and causes an increase in cell death in the outer nuclear layer of the retina, accompanied by significant reductions in the amplitudes of the a- and b-waves of the ERG. The Usher mutation also disrupts localization of the rod and blue cone opsins, further confirming that this is a good model of Usher syndrome [139]. Similarly, CRISPR mutation of *ush2a* increases apoptosis in the outer nuclear layer, and unlike rodent knockouts, there is a significant reduction in a- and b-wave amplitudes, illustrating the usefulness of the cone-dominated retina of zebrafish for modeling certain human diseases [140]. The ERG amplitude is also reduced in a CLCC1 chloride channel knock-out model, which is noted in some inherited forms of retinitis pigmentosa [141]. Similarly, diminished ERG amplitude of the *eyes shut* mutant, another retinitis pigmentosa model, is accompanied by loss of locomotor response to light and mislocalization of both rhodopsin and cone transducin in photoreceptor cilia [142]. The ERG can also be used to confirm functional recovery after acute neurodegenerative changes. For example, the ERG returned to normal about 50 days after ouabain injection, which initially caused extensive structural damage to bipolar cells. The functional recovery was accompanied by increases in dendritic complexity of bipolar cells and their connections to photoreceptors, suggesting that retinal regeneration includes reestablishment of meaningful neuronal connectivity in zebrafish [143].

One important area of study is the effect of diabetes on vision and retinal degeneration. Notable reductions in various ERG parameters including the b-wave amplitude are well established in clinical research as well as basic studies involving rodent and other mammalian species. In wildtype zebrafish, diabetes can be modeled by repeated exposure to 2% elevated glucose in the aquarium water, and this can lead to altered retinal function within about 1 month. Using this approach it was established that hyperglycemic treatment reduced visual-evoked behavior and predominantly affected the blue cones, with apparently selective degeneration compared to rod photoreceptors, determined by histological analysis. The degeneration was accompanied by a significant reduction in the b-wave amplitude, suggesting that the cone degeneration in zebrafish is an early consequence of diabetes and that cones are largely responsible for the b-wave under photopic conditions [144]. Using this approach a more extensive study recorded the ERG generated by isolated eye cups. The amplitude of the native photopic (white light) ERG was found to be significantly lower in hyperglycemic fish compared to mannitol-treated fish, which act as a control for osmotic changes. B-wave parameters were also reduced in response to spectral ERG stimuli, indicating cone-pathway deficits [145]. Hyperglycemia also significantly reduced the amplitude of the d-wave, which is thought to be derived from the OFF-bipolar cells. Recently the pancreatic transcription factor mutant, *pdx1^−/−^* zebrafish was shown to have a diabetic phenotype [146], which exhibits functional deficits including a significant reduction in the b-wave amplitude accompanied by retinal vascular abnormalities and degeneration of rods and RGB cones [147]. The recent data suggest that *pdx1^−/−^* zebrafish may represent a useful new model of retinal complications due to diabetes that include some of the neurodegenerative features leading to loss of visual function. Given the predominance of cone-mediated vision in zebrafish it is likely that this model will contribute to diabetic retinopathy research in ways that have not been possible with the more widely used rodent models.

#### 4.3.2. Ex Vivo Ca^2+^ Imaging of RGCs/Retinal Neurons

Ca^2+^ imaging has been used as an approach to study the neurons of the retina and brain in zebrafish. Expression of GCaMP1.6 was used to demonstrate that long-term potentiation can be elicited in the zebrafish retina at the level of the bipolar cell/RGC synapses and can be induced by visual stimulation [148]. Ca^2+^ levels were also imaged in zebrafish after injection of Oregon Green 488 BAPTA-1, combined with drifting bar visual stimuli, to map the functional directionality of different regions of the optic tectum [149].

Two-photon imaging with zebrafish expressing the Ca^2+^ indicator GCaMP6 under a RGC-specific promotor has been used to identify a specific pretectal nucleus, AF7, which responds to a visual prey stimulus such as paramecia. AF7 is innervated to two specific subtypes of RGCs, suggesting that part of the zebrafish visual system is highly species specific and adapted for prey capture behavior [150]. A similar study that employed Ca^2+^ imaging and optogenetic RGC stimulation showed that the differential responses of approach to small stimuli, representing prey, as opposed to avoidance of large stimuli interpreted as predator, is controlled by AF7 [151]. Two-photon Ca^2+^ imaging in pan-neuronal Tg (elav3:GCaMP5G) zebrafish further established that the behavioral response to a looming visual stimulus (suggestive of an approaching predator) is regulated through broader brain regions in the optic tectum, the pretectal thalamus and the midbrain tegmentum [113].

The *crystal clear* mutant was created to further enhance the ability to image the normally pigmented tissues such as retina, and avoid the use of high toxic concentrations of 1-phenyl-2-thiourea (PTU). This fish carries a combination of mutations affecting pigmentation (*nacre^w2/w2^*, *alb^b4/b4^* and *roy^a9/a9^)* but unlike the *casper* mutant (*nacre^w2/w2^* and *roy^a9/a9^*) these fish lack pigment in the eye and still retain functional vision, as assessed by the OMR. Two-photon Ca^2+^ imaging, using the pan-neuronal GCaMP6f genetic Ca^2+^ indicator reveals apparently normal RGC and optic tectum responses [152]. Thus far, this model has not been used in the context of understanding neurodegenerative changes in the retina.

## 5. Neuroprotection and Regeneration in Zebrafish Retinal Injury Paradigms

Neuroprotection and regeneration are two potential therapeutic strategies that aim to manage the neuronal loss that occurs during neurodegeneration. The former attempts to protect neurons from neurodegenerative damage (Table 1), while the latter intends to replace or rewire lost neurons and synaptic connections, or else induce regrowth of damaged axons (Table 2). The zebrafish retina has the capacity to regenerate when injured, unlike the mammalian retina. As discussed previously, this regenerative response initiates the asymmetric cell division of Müller glia to produce neuronal progenitor cells [86,90,91]. These cells then proliferate and migrate to the site of injury to differentiate into the required neuronal cell type [90,91]. It is also important to note that the inflammatory and immune response play an important role in retinal degeneration, as well as regenerative progression. Recently, microglial cells were shown to be necessary for Müller cell stimulation that allows the retinal regenerative response after laser damage [153]. However, the spinal cord axonal regrowth was unaffected in zebrafish larvae with drastically reduced microglial cells (colony-stimulating factor 1 receptor (csfr1a/b) mutants) [154]. Regeneration studies aim to identify important proteins that may induce or allow for endogenous regeneration in the mammalian retina, therefore replacing or rewiring neurons or axons that are permanently lost due to neurodegeneration [155,156]. Visual neurodegenerative disorders are often diagnosed at later stages of the disease when neuronal loss has already begun, due to visual deficits being detected at that point. Therefore, a combined neuroprotective and regenerative therapeutic approach is a rational strategy for the treatment of retinal neurodegenerative disorders, in order to protect the remaining neurons from further damage while replacing or rewiring neurons and synaptic connections that have been lost. Studies involving neuroprotection and regeneration require the use of neuronal injury paradigms (Table 3). These paradigms simulate neurodegeneration allowing researchers to test effective treatment strategies or decipher disease mechanisms that identify pharmacological- or genetic-based therapeutic targets (Table 1, Table 2, Table 3). To date, there are various retinal neuronal injury paradigms used in zebrafish research. Undoubtedly, the most common of those paradigms is light-induced retinal degeneration [86,90,91,156,157,158,159,160,161,162,163,164,165,166,167,168,169,170,171,172,173,174,175], followed by chemically [95,103,155,176,177,178,179,180,181,182,183,184,185,186] or mechanically induced retinal damage [92,187,188,189,190,191,192,193,194,195,196]. The choice of paradigm depends on the desired outcome, for example, ouabain has a more global retinal degenerative effect (depending on concentration), while intense light exposure primarily affects the photoreceptors [156,169].

### 5.1. Light-Induced Retinal Damage

Light-induced retinal degeneration (LIRD) is one of the most commonly used paradigms in neuroprotection and regeneration studies. The zebrafish usually undergo a period of dark-adaptation, followed by continuous exposure to bright white light leading to photoreceptor damage. The dark adaptation period frequently used is 1–2 weeks, however, it has recently been shown that 24-h is sufficient to render the retina susceptible to light-damage [165]. The effect of continuous light-exposure leads to rhodopsin bleaching and oxidative stress that results in cell death of photoreceptors [173]. One of the earliest papers describing the time course of retinal light-damage in albino zebrafish was by Vihtelic and colleagues [172]. They described widespread rod and cone apoptosis during the first 24 h of constant light, which was followed by cell proliferation within the INL, which they associated with the radial processes of Müller glia. In the study, the rods and cones were replaced within the 28-day recovery period but the well-ordered cone cell mosaic was not re-established [172]. A few years later, the same group embarked on a similar study with albino zebrafish using the LIRD model, where they examined retinas from several time points. Earlier time points were classed as the photoreceptor death stage, middle were the cell proliferation and migration stages, and end stages were marked by the initial photoreceptor differentiation [162]. Following this work, several researchers showed that the LIRD paradigm also caused degeneration in pigmented-zebrafish, expanding its use to the numerous transgenic reporter lines that are available for retinal research [168,171,173]. Thomas and colleagues characterized multiple light paradigms involving different combinations of bright light and UV light in albino and pigmented zebrafish [170,171]. They found that constant bright light primarily damaged rod photoreceptors, meanwhile, UV significantly damaged both rods and cones. The ventral and posterior portions of the retina were naturally protected from light-lesion, however, the combination of UV and constant bright light prevented this neuroprotection. Weber et al. (2013) used pigmented fish to compare retinal lesions between freely swimming fish under diffuse light versus immobilized fish using light focused directly on the retina. Unspecific damage to retinal neurons had occurred at the center of a focused light lesion but not in the diffuse light lesion [173]. Rose Bengal Light activation is another type of LIRD model which has been proposed as a photochemical stress model that can produce retinal lesions, possibly through involvement of the N-Methyl-D-aspartic acid (NMDA) receptor [160].

These early LIRD experiments led investigators to begin identifying the molecular mechanisms underlying photoreceptor and retinal regeneration. Initial investigations by Craig and colleagues performed laser-capture microdissection of the outer nuclear layer (ONL) in LIRD zebrafish retinas in order to analyze the gene expression profile of photoreceptors that die or are regenerated [158]. They validated changes in gene expression and focused on genes encoding growth factors, including galectin-1 like-2 (lgals 1l2) from the galectin family [158]. In a later study, they showed that light-induced photoreceptor death increased secreted β-galactoside binding protein, Galectin 1–like 2 (Drgal1-L2) from microglia and proliferating Müller glia, as well as mitotic progeny [159]. Morpholino knockdown of Drgal1-L2 resulted in reduced regeneration of rods, but not cones, suggesting that Drgal1-L2 may be involved in rod regeneration [159].

Meyers et al. (2012) used genetic and pharmacological manipulation of the β-catenin/Wnt signaling pathway in zebrafish larvae to show that it is required to maintain proliferation in the CMZ and that hyper-stimulation of β-catenin/Wnt signaling inhibits normal retinal differentiation and expands the population of proliferative retinal progenitors [86]. Thomas et al. (2016) were the first to characterize the gliotic potential of zebrafish Müller glia. By inhibiting the Müller glial cell proliferation with intravitreal injections of 5-fluorouracil (5-FU) or proliferating cell nuclear antigen (PCNA) knockdown in photo-lesioned or ouabain-damaged zebrafish retinas, they showed Müller glia can concurrently up-regulate GFAP and re-enter the cell-cycle following retinal injury [156]. Thus, far, there have been various targets identified in playing a role in progenitor cell proliferation using the LIRD paradigm, including Stat-3 [162], miRNAs which reduced INL proliferation at early (*miR-142b* and *miR146a*) or later (*miR-27c* and *miR31*) time points [168] TGF-β signaling via corepressors *Tgif1* and *Six3b* [91], Fgf signaling [161], Rock2a and Rock2b [166], Wnt signaling [86], human epidermal growth factor receptor 4 (Her4) [174] and Capn5 [157].

Several promising therapeutic targets have been identified using the zebrafish LIRD model. LIRD injury has been shown to decrease when given 5-fluoro-uracil, a nuclear factor erythroid 2-related factor (Nrf2) activator, or ciliary neurotrophic factor (CTNF) [156,163,197]. Fibroblast growth factor receptor 1 (FGFR1) signaling also protects the rods from light damage and induces rod precursor proliferation [167]. Following comparative transcriptome analysis in rodents exposed to light-induced retinopathy, Kawase et al. (2016) inhibited the histone acetyltransferase, EP300, a key upstream regulator of cytokine signaling axis, and found increased retinal cell apoptosis, decreased photoreceptor outer segments and increased proliferation of Müller cells following light damage; suggesting the EP300 may protect photoreceptor cells from light-induced retinal damage [164].

**Table 1:** Summary of retinal neuroprotection studies that use zebrafish retinal injury paradigms.

Retinal Injury Paradigm	Model	Age	Neuroprotective Agent or Mechanism	Reference
Light-Induced				
LIRD	Retinal degeneration	Larvae	EP300 (Histone acetyltransferase)	[164]
LIRD and ouabain	Retinal degeneration	Adult	SHH-N recombinant protein	[169]
Rose Bengal Light lesion	Retinal degeneration	Adult	Thiokynurenate (NMDA inhibitor)	[160]
Mechanical				
Optic nerve injury	RGC loss/injury	-	Neuroglobin	[195]
Chemical-Induced				
NMDA-induced neurodegeneration	Retinal degeneration	Adult	Resveratrol and MK-801	[180]
	Glaucoma	Adults	Resveratrol	[185]
Acrylamide toxicity	Retinal Toxicity	Embryo	Carnosic acid	[176]
*6-OHDA*	Night blindness	Larvae/adult	Stil-mediated Shh signaling	[179]
Oxidative Stress				
Hypoxia/reperfusion	Retinal degeneration	Embryo	HSF1	[198]
Hypoxia	Hypoxia-driven retinal angiogenesis	Adult	Sunitinib and ZN323881 (anti-VEGF drugs)	[199]
Hydrogen peroxide	RGC degeneration	Larvae	Neurotrophins-magnetic nanoparticles	[200]
Paclobutrazol	Hypoxia	Embryo	Retinoic Acid	[186]
Age	Age-related oculopathy	Adult	Resveratrol	[201]
Diet-Induced				
MeHg-diet exposure	Retinal Toxicity	Embryo	Selenium	[95]
Genetically Targeted				
von Hippel-Lindau mutants	Vascular-driven retinopathies	Embryo	Sunitinib and 676475	[202]
*Gdf6* zebrafish mutants	Early onset retinal dystrophies	Embryo	Aminopropyl Carbazole, P7C3	[203]
AMD, Tg(rho:hsa.HTRA1); RP, Tg(rho:hsa.RHO_Q344ter)	AMD and RP	Larvae	6-boroV (HTRA1 inhibitor)	[204]
Tg line *dyeucd6*	RP	Larvae	Tubastatin A (TST)	[205]

Abbreviations: LIRD, light-induced retinal degeneration; HSF, Heat shock factor; Shh, Sonic hedgehog; RGC, retinal ganglion cells; MeHg, Methylmercury; SeMet, selenomethionine; 6-OHDA, 6-hydroxy-dopmine; HTRA1, High-Temperature Requirement A 1; RP, retinitis pigmentosa; AMD, age-related macular degeneration.

**Table 2:** Summary of retinal regeneration studies that use zebrafish retinal injury paradigms.

Retinal Injury Paradigm	Retinal Model	Age	Regenerative Target or Mechanism	Reference
Light-Induced				
LIRD	Retinal degeneration	Adult	Shh signaling	[169]
			Several miRNAs	[168,206]
			TGFβ signaling	[91]
			β-catenin/Wnt signaling	[86]
			Identified markers for stages of regeneration	[87]
			Reported numerous gene expression profiles	[162]
	Photoreceptor degeneration	Adult	Rho-associated coiled-coil kinase 2 (a and b)	[166]
			FGF signaling	[161]
			Drgal1-L2 secretion	[159]
			Reported numerous gene expression profiles	[158]
		Adult/Larvae	Capn5	[157]
		Adult/Larvae/Embryo	Her4 expression	[174]
Laser Focal injury	Retinal injury	Adult	Microglia and Müller cell signaling	[153]
Mechanical, Light and Chemical retinal lesions	Retinal degeneration	Adult	Müller glia-derived progenitors	[193]
Retinal lesions and UV light damage	Retinal degeneration	Adult	Jak/Stat signaling and MG reprogramming	[175]
Mechanical				
Retinal stab injury	Retinal degeneration	Adult	Granuin 1	[196]
			Wnt signaling and GSK-3β inhibition	[194]
Retinal stab injury and optic nerve crush	Retinal degeneration	Adult	α1 Tubulin-expressing Muller glia	[92]
Rod photoreceptor ablation and retinal puncture	Age-related oculopathy			
Optic nerve injury	Oxidative stress	Adult	Neuroglobin	[189]
	RGC axon degeneration	Adult	Leukemia inhibitory factor	[192]
	RGC loss/injury	-	Neuroglobin	[195]
Optic nerve crush	Optic nerve degeneration	Adult	Acute inflammatory response	[207]
	Optic nerve injury	Adult	zRICH protein	[187]
		Larvae/Adult	Calretinin expression	[188]
Chemical-Induced				
Intravitreal injections of ouabain	Retinal degeneration	Adult	Microglia and the immune system	[182]
			Purinergic signalling	[181]
			Protemic profiles reported	[155]
			Surviving Neurons	[208]
			ADP	[177]
Light-damage and ouabain injections	Retinal degeneration	Adult	N-cadherin	[89]
Geneticcally Targeted				
*Pde6c^w59^* mutants	Photoreceptor degeneration	Adult	Rip3 Kinase signalling	[209]
		Embryo	Schisandrin B	[210]
Cell-specific ablation	Rod photoreceptor ablation and retinal puncture	Larvae/Adult	Microglial signaling	[211]
	RPE ablation	Larvae/Adult	Wnt Signaling	[212]
	UV cone ablation	Larvae	H3 horizontal cells	[213]

Abbreviations: LIRD, light-induced retinal degeneration; TGF, Transforming growth factor beta; FGF, fibroblast growth factor; Shh, Sonic hedgehog; Drgal1-L2, β-galactoside binding protein Galectin 1–like 2; Capn5, Calpain-5; ADP, adenosine diphosphate; RIP3, receptor-interacting protein kinase 3; UV, ultraviolet; RICH, Regeneration Induced CNPase Homologues; Her4, Hairy-related 4.

**Table 3:** Studies characterizing retinal neurodegeneration and/or regeneration in zebrafish retinal injury paradigms.

Neuronal Injury Paradigm	Model	Age	Reference
Light-Induced			
LIRD	Photoreceptor degeneration	Adult	[165,170,172]
Focused Light lesion	Retinal degeneration and regeneration	Adult	[129,173]
Mechanical			
Optic nerve crush	Optic nerve injury	Adults	[190]
	Optic nerve remyelination	Adults	[191]
Chemical			
Ouabain	Inner retinal neuron regeneration	Adults	[88,143,214]
Acrylamide toxicity	Photoreceptor degeneration and regeneration	Adults	[178]
N-methyl-Nnitrosourea	Photoreceptor degeneration and regeneration	Adults	[103]
Cypermethrin	Retinal Toxicity	Adult	[183]
Diet-induced			
SeMet-diet exposure	Retinal toxicity	Adult/Embryo	[184]
Glucose immersion	Diabetic Retinopathy	Adults	[144,145,215]
	Gestational hyperglycemia	Embryo	[216]
Genetically Targeted			
ZF*cerkl* morpholino knockdown	Retinal dystrophies	Larvae/Adults	[217]
Tg lines *Pde6c^w59^* and *Xops*:mCFP*^q13^*	RP and cone-rod dystrophy	Embryos/Adults	[218]
*pcare1^rmc100/rmc100^*	Diabetic Retinopathy	Adults	[219]
Tg *bugeye* mutants	Glaucoma	Larvae	[106,220,221]
NTR/MTZ Cell specific ablation	Rod photoreceptor ablation	-	[222]
	Cone photoreceptor ablation	Larvae	[104,223]
	Bipolar Cell ablation	Larvae	[224,225]

Abbreviations: LIRD, light-induced retinal degeneration; SeMet, selenomethionine; NTR, Nitroreductase; MTZ, metronidazole).

### 5.2. Mechanical Retinal Damage

Two major retinal injury paradigms use mechanical force to cause neuronal injury, namely, retinal stab injury [175,193,194,196] and optic nerve crush (or injury) [188,190,192,207]. The former commonly uses a needle to stab the retina in a uniform manner, while the latter uses forceps to crush, or scissors to transect the optic nerve, creating a lesion. The retinal stab injury is usually used to model a degeneration in all layers of the retina. Interestingly, Powell et al. (2016) used a combination of mechanical, light and chemical-induced retinal lesions to show that Müller glia-derived progenitors regenerate all major retinal cell types regardless of which neuronal cells were damaged. New cells were found in undamaged areas as well as the damaged areas, however there was greater proliferation in the damaged areas, suggesting that there is feedback inhibition from surviving neurons that may suppress localized regenerative mechanisms [193].

#### 5.2.1. Retinal Stab Injury

Retinal stab injury paradigms have mostly been used for regeneration experiments in zebrafish, but have the potential to be used as the neuronal injury paradigm in neuroprotection assays. There are several signaling pathways involved in regeneration, mostly involving the *ascl1a* gene, which encodes a nodal transcription factor impacting reprogramming genes and signaling cascades that affect almost all aspects of retinal regeneration [175]. Retinal injury-induced expression of Asc1a has been shown to be regulated by Jak/Stat signaling pathway, which can be activated by Leptin and cytokines [175], and it can also suppress Wnt signaling inhibition, suggesting that Ascl1a expression contributes to the multipotential character of the progenitors [194]. Furthermore, Tsuruma et al. (2018) showed granulin 1 (grn1) was increased after retinal injury, whereby knocking out the gene reduced the proliferation of Müller cells undergoing regeneration. Following intravitreal injection of recombinant grn1, *asc1a* and *lin28* were expressed, which promoted Müller cell regeneration, thus suggesting a potential therapeutic target to stimulate dedifferentiation of Müller glia and promote retinal regeneration [196].

#### 5.2.2. Optic Nerve Crush/Injury

RGCs are the only cells in the retina that send visual information to the brain via their axons. The axons are bundled and myelinated via the optic nerve, where they project to and terminate at specific brain regions. The optic nerve is a simple axonal system that is, certainly, a popular model used for axonal regeneration and remyelination studies. Optic nerve injury models, such as optic nerve crush, are used in several species, particularly in zebrafish due to their high regenerative capacity. Following optic nerve crush, almost all RGCs survive and are able to regenerate [226,227,228]. The RGCs upregulate growth-associated genes from 1 day till 4 days post injury [226,229]. RGC axons regrow and reinnervate the tectum between 5 and 14 dpi [230,231], and visual function occurs by 20–25 dpi [226,228,231,232]. An earlier study from 2004 showed that calretinin, a Ca^2+^-binding protein involved in Ca^2+^ buffering and neuroprotection, was downregulated in RGCs up to 10 days post-optic nerve crush, where levels returned to normal by 13 days post-injury at the same time axons were regenerating; suggesting an increase in cytosolic Ca^2+^ is essential for axon outgrowth [188]. Several years later, a study used optic nerve crush injury to show that GFAP should not be used solely as an astrocyte marker during optic nerve regeneration in zebrafish due to lack of expression, they showed that cytokeratin expression alongside GFAP should be used instead [190]. Interestingly, Ogai et al. (2014) showed that leukemia inhibitory factor (LIF), is upregulated in zebrafish RGCs at 3 days post injury (dpi; optic nerve crush), and that activation of signal transducer and activator of transcription 3 (STAT3), a downstream target of LIF, occurs at 3–5 dpi. LIF knockdown using LIF-specific antisense morpholino oligonucleotides via a severed optic nerve, reduced the expression of LIF and, therefore, prevented the injury-induced activation of STAT3 in RGCs as well as other detrimental effects on axonal regeneration. This suggested that the upregulated LIF drives Janus kinase (Jak)/STAT3 signaling in zebrafish RGCs after nerve injury, highlighting the role of LIF and offering a possible therapeutic target for optic nerve regeneration [192].

Macrophage/microglial response has been suggested as a possible target for optic nerve regeneration and remyelination. Munzel et al. (2014) developed a novel method to induce a focal demyelinating lesion in the optic nerve of adult zebrafish, in order to observe remyelination and cellular changes over a course of time. They found that, despite the internodes being short, the number of myelinated axons was restored to normal at 4 weeks following optic nerve injury in both young and old zebrafish, however, the myelin sheaths in the old zebrafish were thinner than normal and remained so for 3 months. This age-related thinness in remyelination was associated with a reduced macrophage/microglial response [191]. A recent study found a strong positive correlation between the retinal inflammatory response and regenerative capacity of the optic nerve in adult zebrafish following optic nerve crush [207]. The corticosteroid, dexamethasone (dex), was shown to suppress regeneration after optic nerve crush injury and sensitize retinal microglia [207].

### 5.3. Chemical-Induced Retinal Damage

There are multiple agents commonly found in pesticides, fungicides, and water treatment chemicals that are toxic to zebrafish retinal development. Acrylamide, shown to induce oxidative stress and thinning in the photoreceptor layer, is one such product [178]. The effects of acrylamide were attenuated using carnosic acid, whereas paclobutrazol activity was neutralized by administering retinoic acid [176,186]. Common components of pesticides, such as paclobutrazol and cypermethrin, were also reported to cause cell death in the photoreceptor layer [183,186]. Elevated intraocular concentrations of NMDA may play an important role in RGC loss, a major characteristic in glaucoma and retinal neurodegeneration [180]. Luo et al. (2019) delivered NMDA to adult zebrafish in three ways: immersion, intravitreal injection and intraperitoneal injection, and found intravitreal injections to be the most appropriate for retinal damage. They showed significant apoptosis in the ganglion cell layer (GCL) and reduction in retinal layer thickness, whereby administration of the NMDA receptor antagonist MK801 and natural product, resveratrol, prevented NMDA-induced retinal neurodegeneration [180]. Intravitreal injections of the neurotoxin, ouabain, has been described as a model of inner retinal cell death in the adult zebrafish retina, sparing the photoreceptor and Müller glia [88,233]. Though, ouabain concentration can be scaled to trigger death of only inner retinal neurons or all retinal neurons [88,143,208,214]. Bipolar cells that were regenerated following ouabain-induced chemical lesions were fewer in numbers, but the rewired circuitry of the regenerated neurons was shown to be accurate [143]. In ouabain-injured RGCs, research has shown that adenosine diphosphate (ADP) plays a role in retinal recovery by increasing proliferation [177]. Proteomic expression profiles during degenerative and regenerative stages in adult zebrafish have identified several key proteins to target in the retina following retinal degenerative disease, including galectin-1, cytoskeleton and membrane transport proteins [155]. More recently, Mitchell et al. (2018) documented an early leukocyte infiltration response to ouabain injection in the retina, followed by immune cell proliferation (likely microglia and macrophages), following which the Müller glia re-enter the cell cycle [182]. Medrano et al. (2017) showed that specific purinergic signals are upregulated following ouabain retinal injury. Interestingly, the extracellular nucleotide agonist, ADPβS, induced the expression of *lin28a* and *ascl1a* genes in mature regions of uninjured retinas. This gene expression was inhibited by blocking the early injury-induced activation of P2RY1, which also prevented progenitor cell proliferation [181]. Sonic hedgehog (shh) was also found to be upregulated after ouabain injury, and treating the retina with shh resulted in Müller cell hypertrophy followed by their conversion to retinal progenitor cells [169]. Additionally, the presence of surviving neurons helped to facilitate the regeneration process by regulating factors such as Shh signaling [208]. In a retinal excitotoxicity model, inhibition of shh has been shown to make retinal dopaminergic interplexiform cells (DA-IPCs) more susceptible to excitotoxicity induced by 6-hydroxydopamine (6-OHDA) [179]. Maurer et al. (2014) established a retinal injury protocol using the chemical MNU to induce retinal damage in adult zebrafish. Visual acuity of these fish was measured using the optokinetic reflex, and was found to be decreased 3-days post treatment, returning to baseline levels after a further 3 days. Histological deficits in this study followed a similar timeline [103]. N-cadherin-mediated adhesion has been shown to be necessary for both neurogenic cluster formation and neuronal progenitor migration to regenerate neurons following oubain-induced retina injury in zebrafish [89].

### 5.4. Oxidative Stress-Induced Retinal Injury

There are multiple ways to increase oxidative stress in the retina, including creating a hypoxic environment by injecting H_2_O_2_. In a hypoxic environment, angiogenesis occurred in the zebrafish retina via VEGF2 receptor activation, which is very similar to the response in human eyes [199]. Injecting H_2_O_2_ results in cell injury in the GCL, and nanoparticle delivery of neurotrophins was protective against oxidative stress [200]. Chimeric neuroglobin was also shown to protect against oxidative stress, and treating zebrafish retinal explants with neuroglobin enhanced neurite outgrowth [189,195]. Neuroglobin expression also increased in the IPL and INL when the optic nerve was injured, as did regeneration-induced 2′,3′-cyclic-nucleotide 3′-phosphodiesterase homologues in the RGCs [187,189]. 

A low tissue oxygen concentration, followed by the reintroduction of oxygen, gave rise to a phenomenon known as hypoxic/reperfusion injury [198]. Tucker and colleagues (2011) preconditioned zebrafish embryos to heat-shock treatment prior to hypoxic/reperfusion (HR) exposure. This consisted of immersing the heat-shock preconditioned embryos in hypoxic medium for various times followed by incubation in oxygenated medium [198]. They found that heat shock preconditioning and/or HR elevated the expression of heat shock proteins and uncoupled the role of HSF1 to HSP expression.

### 5.5. Diet-Induced Retinal Damage

Methylmercury, a toxin that can accumulate in fish and cause issues in the people that consume them, has been shown to impact the vision of the zebrafish and cause thinning in the INL of the retina [95,234]. Some of these toxins, such as selenium, can cause damage to the progeny, resulting in visual impairment [184]. Similarly, glucose immersion decreases the ability of blood vessels to support the retina by thickening the walls of these vessels. Gleeson et al. (2007) were one of the first groups to develop a hyperglycemia model that induces retinal damage in zebrafish. This, and most other glucose immersion studies, oscillate zebrafish between high glucose-water and fresh-water every 24-h for a set period of time; usually around 30 days [144,215]. Alvarez et al. (2010) characterized a novel model of non-proliferative diabetic retinopathy in adult zebrafish by inducing hyperglycemia using this oscillating method. They found visual function, cone morphology and function, and blood-retinal barrier integrity, were all diminished in hyperglycemic fish [144]. Singh et al. (2019) used a similar glycemic oscillation paradigm to study the effect of gestational hyperglycemia on the developing embryonic retina. Embryos at 3 hpf were alternated between glucose-water (at various concentrations) and fresh water every 24-h until 5 dpf. The embryos exposed to high glucose exhibited a number of morphological retinal defects, including differences in retinal cell layer thickness, increased macrophage numbers, and decreased numbers of Müller glia and RGCs. Glucose exposure is detrimental to the development of embryonic retina and the legacy of this exposure may extend into adulthood [216].

### 5.6. Genetically Targeted Retinal Damage

A major benefit of using zebrafish in research is that they are easy to genetically manipulate. For this reason, zebrafish are popular in neuroprotection and regeneration studies, as these genetic manipulations can be used to either develop a retinal injury/degenerative model, or to develop potential gene therapy targets. There are zebrafish models of inherited vision loss, such as the XOPS, Pde6e, and CERKL mutant zebrafish. XOPS fish have rod degeneration, while pde6e mutant fish have cone degeneration. The bipolar cells will reorganize themselves in response to cone degeneration and there are also decreases in glutamate receptor expression. This effect is attenuated when rods are induced to proliferate [218]. Pde6e mutant zebrafish embryos were treated with Schisandrin B (SchB), an active component from the fruit, Fructus Schisandrae, which is believed to possess prophylactic visual benefits. SchB-treated zebrafish appeared to display morphological and functional rod improvement, but did not rescue dying cones [210].

When the rods are lost, her4, a part of the Notch-delta signaling pathway, is upregulated in the CMZ and a sub-set of Müller cells. Her4-positive CMZ cells become retinal neurons and Müller cells, with the exclusion of rods, and her4-positive Müller cells contribute to rod regeneration [174]. CERKL mutations, which can cause severe retinal dystrophy in humans, are involved in lamination of the retinal layers and lens development. Although knocking down CERKL resulted in increased cell death, it did not seem to be necessary for proliferation or early differentiation [217]. HTRA1, a protein shown to be involved in the pathophysiology of age-related macular degeneration, has been shown to induce an accumulation of lipofuscin and melanolipofuscin between the photoreceptor and retinal pigment epithelial layers when overexpressed. When HTRA1 is overexpressed, TGF-beta/Akt pathway activity also increased, resulting in FOXO1 phosphorylation and photoreceptor cell death [204]. HDAC6 is another protein shown to be overexpressed in retinal degenerative diseases. When inhibited, the retinal morphology and cell survival improved in an inherited blindness model [205]. Mutations of the von Hippel-Lindau (VHL) tumor suppressor gene in zebrafish mutants display a marked increase in blood vessel formation throughout the embryo, which correlated with areas of high vascular endothelial growth factor (VEGF) mRNA expression in the eye. The vhl^−/−^ retina exhibited vascular leakage, severe macular edema and retinal detachment. Sunitinib and 676475 were used to inhibit the VEGF receptor (VEGFR) tyrosine kinase, which prevented vhl^−/−^-induced angiogenesis. The group reasoned that this genetic model is a cost-effective and non-invasive method for screening for novel pharmacological agents and combinatorial treatments [202]. Asai-Coakwell and colleagues (2013) showed that mutations in the transforming growth factor-b (TGF-b) ligand, Growth Differentiation Factor 6 (Gdf6), gdf6 results in photoreceptor degeneration and retinal apoptosis. P7C3, a novel aminopropyl carbazole, rescued gdf6-deficient zebrafish embryos from retinal apoptosis, suggesting that TGF-b signaling is mechanistically involved in retinal dystrophies and could be a target for gene therapy [203]. Recently, a stable pcare1 mutant zebrafish model was generated by disrupting the coding sequence using CRISPR/Cas9 technology, which caused retinal disorganization of photoreceptor outer segments and impairment in visual functional in embryos and larvae [219]. The *Bugeye* zebrafish mutant, possessing a mutation in the *low density lipoprotein receptor-related protein 2 (lrp2)* was identified and proposed as model for myopia and glaucoma. Lrp2 is an endocytic receptor for several bioactive molecules including Sonic hedgehog, Bone morphogenic protein 4, retinol-binding protein, vitamin D-binding protein, and apolipoprotein E [221]. Bugeye mutants develop high IOP, enlarged eye globes [221] morphological abnormalities and functional deficits in the retina [106]. Their retinal cells have also been shown to proliferate in response to cell stress whereas the WT cells would die [106,220].

Death of specific retinal cell types can be achieved by using transgenic zebrafish with bacterial nfsB/NTR under a cell specific promotor, which results in ablation of the designated cells following Metronidazole (Mtz) administration [222]. Using UV cone promotors, it was shown that targeted cell death was specific to UV cones as neighboring cells were not injured following Mtz ablation; also, horizontal cells had reestablished connections with UV cones within a certain time window [213]. Blue cones were found to regenerate faster than UV cones after ablation, and rod photoreceptors play a role in replenishing the numbers of the UV cones [104,223]. When rods are ablated, Müller cells and other innate immune cells play a role in rod regeneration in the zebrafish retina [211]. Other retinal cells, such as bipolar cells and RPE, have also been ablated and their regeneration patterns were studied. Zebrafish are capable of regenerating RPE cells, which involves the Wnt pathway, in a peripheral to central manner [212]. Bipolar cells regenerate seven days after ablation and are able to reestablish most of the connections [224,225]. This genetic tool has shown that most cells in the zebrafish retina have the capability to be regenerated, which may be useful in gaining more insight into retinal repair.

A final interesting study, worth mentioning here, is one recently published by Didiano et al. (2020), who isolated extracellular vesicles (EVs) from C6 glioma cells and injected them into the vitreous of zebrafish. This caused an increase in Müller glia-derived proliferating cells. Morpholino knockdown of Asc1a expression prevented the EV-induced response, suggesting that the EVs may act via Ascl1a, a required transcription factor for zebrafish retinal regeneration [235]. EVs are small vesicular bodies released from cells as a form of cell–cell communication, used in both a locally and distant autocrine or paracrine fashion [235]. Here, the researchers proposed that the glial origin of C6 cells may produce EVs with membrane and cargo content that target Müller glia. Identifying the membrane and cargo content of EVs in various cellular environments, i.e., healthy vs. diseased, is becoming increasingly popular amongst researchers, as they are believed to be a key factor in disease mechanisms and progression, and may have potential therapeutic value. Administration of potentially neuroprotective or regenerative EVs following retinal injury will be a novel and innovative approach to treat retinal neurodegenerative disorders in the future.

## 6. Discussion and Conclusions

Zebrafish offer a convenient animal model for retinal neuroprotection and regeneration research due to their size, economical value and accessibility to genetic manipulation. There are several functional assays available as useful endpoints in neurodegeneration and retinal injury studies. Additionally, the zebrafish retina has several advantages over the rodent in modeling photopic color vision similar to humans. During retinal degeneration or injury, neurons and axons of the retina can be damaged or undergo cell death. The mammalian CNS neurons have little to no regenerative capacity, however, this capacity is high in the zebrafish. Identifying the key players involved in the mechanism of regeneration in zebrafish may lead to the induction of regeneration in the mammalian retina, therefore replacing permanently lost neurons following retinal injury. Additionally, protection of surviving neurons following degeneration or injury is long sought after. The major targets identified for retinal neuroprotection and regeneration using zebrafish include signaling pathways such as: neurotrophic [100,163]; other growth factors [91,158,161,167,174,202,203]; microglia/macrophage [159,182,191,207,216]; Jak/Stat [162,175,192]; Wnt [86,194,212]; Shh [169,179] and NMDA receptor-mediated signaling [160,180]. There is currently limited use of the zebrafish compared to more popular rodent models, but the trend towards utilizing this unique animal model is likely to increase over time and lead to exciting new developments in retinal neuroprotection and regeneration.
